# THROUGH THEIR LENS: A PHOTOVOICE STUDY OF OLDER ADULTS’ EXPERIENCES OF ACCESSIBILITY AND AGING IN COMMUNITY

**DOI:** 10.1093/geroni/igad104.3757

**Published:** 2023-12-21

**Authors:** Yeonjung Lee, Lauren Migrino, Rochelle Deloria, Kurt Robertson

**Affiliations:** Chung-Ang University, Seoul, Republic of Korea; University of Calgary, Calgary, Alberta, Canada; University of Calgary, Calgary, Alberta, Canada; University of Calgary, Calgary, Alberta, Canada

## Abstract

The objective of this research is to explore the lived experiences and perceptions of older adults in relation to aging in community and accessibility to social, physical, and community resources. Photovoice was used as the methodology for this study so that older adults could take pictures of people, places or things that might shed light on what is most important to them and their experiences of accessibility and aging in their respective community. Findings present qualitative themes with thirteen older adults in seven neighbourhoods in Calgary, Canada including planning for the future; convenient access to resources and services; importance of ethno-cultural supports and diversity; affordability; and importance of cultivating connection. These themes demonstrate that aging in place requires older adults to proactively anticipate and plan for the aging process. Moreover, aging in community for older adults means having access to physical resources that are convenient and in close proximity. Additionally, improving access to ethno-cultural resources and language supports enhances older adults’ sense of safety and belonging. Older adults highlight an importance of having a diverse group of community members encompassing different ages, ethno-cultural backgrounds, and ability levels. Affordable resources and supports are essential for older adults to maintain a fulfilling and independent lifestyle within their built and social environments. Lastly, older adults seek social connection and belonging, but limited access to social supports can lead to social isolation and disconnection from their community.

